# Data needed for assessing water footprint of steel production

**DOI:** 10.1016/j.dib.2020.105461

**Published:** 2020-03-20

**Authors:** Reza Nezamoleslami, S. Mahdi Hosseinian

**Affiliations:** Department of Civil Engineering, School of Engineering, Bu-Ali Sina University, Iran

**Keywords:** Water footprint, Energy consumption, Steel industry, Raw materials

## Abstract

The data presented in this paper offer the information needed for assessing the water footprint of steel production. The data provided here are related to the research paper, “An Improved Water Footprint Model of Steel Production Concerning Virtual Water of Personnel: The Case of Iran” [1]. The data were collected form analysing a case study and reviewing the relevant literature. The case study data were collected from a large steel plant in Iran. All information received from this plant is related to 2017. The primary data were collected by interviewing the head of the research and development of the plant. All relevant formal documents of the plant and information on its web site were also reviewed. For data related to the water footprint of energy and raw materials, used for steel production, the relevant studies were reviewed.

Specifications Table

**Table:** 

Subject	Water scarcity
Specific subject area	An improved water footprint model of steel production
Type of data	Table
How data were acquired	A steel plant manufacturing various industrial and constructional steel sections, operating based on a blast furnace method, was analysed. The primary evidence was collected through interviews with the head of the research and development (R&D) of the plant. The interviews were conducted several times and structured to ensure that key parameters related to the model boundary were covered. All relevant formal reports of the plant and information on its web site were reviewed. Also, several visits were undertaken to the steel plant. The visits provided an opportunity for informal conversations with the operational level personnel responsible for different sections and understanding the processes followed at the site. Such conversations helped in the triangulation of information received from different sources, which ensures the validity and reliability of the data [2]. The findings were also verified against existing literature to identify similarities and differences. All information about water, energy, electricity, and other fuels consumption in the plant as well as steel production are related to 2017. For the data from the literature, relevant studies related to the water footprint of mining and processing of steel raw materials and various types of energy were reviewed.
Data format	Raw data
Parameters for data collection	The parameters for data collection are based on the water footprint network methodology. The attention is given to the blue water footprint to refer to surface water or groundwater used for steel manufacturing [3].
Description of data collection	For direct water (DW) and energy consumptions, three main parts are considered: (1) the production line (blast furnace, converter, ladle furnace, casting and rolling sections); (2) raw material processing (coke, agglomeration, pelletising, and lime); and (3) facilities and washing. For virtual water consumption, data related to: (1) electricity generation; (2) natural gas extraction and processing; (3) coal mining; (4) coke processing; (5) iron ore mining; (6) limestone mining; (7) transportation; and (8) personnel are presented.
Data source location	A steel plant, marked as the largest constructional steel maker in Iran, with 3.6 million tons capacity per year, is the source of data collection.
Data accessibility	With the article
Related research article	Nezamoleslami, Reza, and S. Mahdi Hosseinian. An improved water footprint model of steel production concerning virtual water of personnel: The case of Iran. Journal of Environmental Management, 260 (2020): 110,065.‏ https://doi.org/10.1016/j.jenvman.2020.110065[1]

## Value of the data

• The data are important as they provide an independent reference for steel's water footprint assessment.
• Steel's manufactures might benefit these data in assessing their dependence on water resources particularly for developing the current steel plant and for finding the location of a new plant.
• Researchers might use these data to compare their findings on water footprint of steel production in different countries with different technological routes.
• Researchers might also use these data to evaluate the impact of energy and employees on the water footprint of steel production.
• The data help society think about the water footprint of steel production in the short term and develop a sustainable steel production program in the long term.

## 1 Data

The data set provided in this section consists of two parts. The first part is related to the steel plant. The second part is related to the water footprint data of energy and raw materials, used in steel plants, collected from the literature.

### 1.1 Case study data

Table 1 presents the data collected from the selected steel plant in three categories: (1) general; (2) employees; and (3) energy and raw materials used in the plant.

**Table 1:** The case steel plant data.

Category	Parameter	Values
General information	Production of steel	2.9 × 10^6^ ton
	Plant's direct water consumption in the production line	20 × 10^6^ m^3^
	Water losses in the plant	1%
	The distance to the nearest center of population	35 km
Employees information	Number of employees	15,000 people
	Number of employees in a work shift	5000 people
	Working days per week	6 day
	Number of shifts in a day	3
	Hours of a work shift	8 h
	Diesel fuel vehicles travel for transferring employees	375
	Each employee's water consumption in a work shift	30 L
Energy and raw materials	Electricity generated by the thermal power plant	1.020 × 10^6^ MWh
	Coke consumption (supplied from outside the plant)	5.300 × 10^5^ ton
	Coke consumption (produced in the plant)	7.200 × 10^5^ ton
	Natural gas	7.320 × 10^8^ m^3^
	Coal	1.050 × 10^6^ ton
	Iron ore	5.022 × 10^6^ ton
	Limestone	4.455 × 10^5^ ton

### 1.2 Literature data

The data obtained from the literature comprise water footprints of: (1) natural gas mining and processing; (2) electricity generation; (3) coal mining; (4) iron ore mining; (5) limestone mining; (6) transportation (diesel-based vehicles); and (7) employees' meal. Table 2 presents the water footprint data of these parameters based on the best sources available in the literature.

**Table 2:** Water footprint data of different parameters.

Parameter	Water footprint	Sources
Natural gas mining and processing	9.251 × 10^−3^ (m^3^/m^3^)	[4,5,6,7]
Electricity generation	1.8 m^3^/MWh	[5,6,7]
Coal mining	0.36 (m^3^/ton)	[8,9]
Coke processing	1.03 (m^3^/ton)	[9]
Iron ore mining	0.425 (m^3^/ton)	[10]
Limestone mining	1.09 (m^3^/ton)	[11]
Transportation (diesel-based vehicles)	0.22 × 10^−3^ (m^3^/km)	[12]
Employees' meal	4.757 (m^3^/meal)	[6,7,13]

## 2 Experimental design, materials, and methods

The experimental design, materials, and methods are based on a model boundary and a proposed water footprint model from [1]. Table 3 presents focus areas for assessing water footprint of steel production. For direct water consumption, three main parts were considered: (1) the production line (blast furnace, converter, ladle furnace, casting and rolling sections); (2) the power plant and raw material processing (coke production, agglomeration and pelletizing, baked lime production and chemical sections); and (3) facilities and washing. For virtual water consumption, data related to (1) raw material (coal, iron ore and limestone) mining; (2) energy; and (3) employees' food were collected. The electricity of the steel plant is assumed to be generated by power plants located in the plant.

**Table 3:** Focus areas for assessing water footprint of steel production.

Direct water	Production line	Blast furnace
		Converter
		Ladle furnace
		Casting and rolling sections
	The power plant and raw material processing	Coke production
		Agglomeration and pelletizing
		Baked lime production
		Chemical sections
	Facilities and washing	
Virtual water	Raw material mining	Coal
		Iron ore
		Limestone
	Energy	Natural gas
		Coke
		Transports
	Employees' food	

## Conflict of Interest

There is no conflict of interest.
